# NAVIGATING STREET AND SHELTER LIFE: THE CONSEQUENCES OF FORCED MOBILITY FOR OLDER HOMELESS WOMEN

**DOI:** 10.1093/geroni/igz038.655

**Published:** 2019-11-08

**Authors:** Judith G Gonyea

**Affiliations:** Boston University, Boston, Massachusetts, United States

## Abstract

Older homeless women have largely been an invisible population. Two co-occurring trends however are bringing them into the public spotlight—the aging and the feminization of the adult homeless population. Yet despite the steep increase in their numbers, relatively little research exists about how gender and age intersect to shape the homeless experience. Such information is critical if we are to transform our nation’s homeless system, which is based largely on a male model of homelessness, to better support women at risk or experiencing homelessness. In this presentation, we therefore share findings from our qualitative study of homeless older urban women. Using a phenomenological approach, we conducted and recorded semi-structured, in depth interviews with fifteen chronically homeless women in their fifties. Our analysis process was inductive and iterative with the culminating phases being the generation and interpretation of themes. Our analysis revealed the links between place, social connection, sense of belonging, and identity. The women’s narratives uncovered how the time-space discontinuity, created through homelessness, shaped the struggles they faced in trying to survive in degraded or threatening environments, altering their identities and impacting self-esteem. Also revealed was that mobility is a key factor to maintaining the place-identity connection. The women’s narratives highlighted how forced mobility with constrained choice not only led to their pathways into homelessness but also dominated their daily navigation of street and shelter life. We conclude by exploring the question of how we might redesign policies and programs to disrupt homelessness for women in later life.

